# Asymmetry to Symmetry: A Case Report of Mandibular Asymmetry Managed With Distraction Osteogenesis Followed by Genioplasty

**DOI:** 10.7759/cureus.106032

**Published:** 2026-03-28

**Authors:** Subhasish Burman, Asish K Das, Purbalee Barman, Abhishek Khatua, Swagatam Samanta, Moumita Ghosh

**Affiliations:** 1 Oral and Maxillofacial Surgery, Dr. R. Ahmed Dental College and Hospital, Kolkata, IND

**Keywords:** distraction osteogenesis (do), facial symmetry, genioplasty, mandibular asymmetry, mandibular hypoplasia

## Abstract

Mandibular asymmetry is a facial deformity that affects both function and appearance. It may result from developmental disturbances, trauma, or temporomandibular joint pathology. Distraction osteogenesis (DO) offers a biological method for gradual bone elongation along with simultaneous adaptation of surrounding soft tissues. Genioplasty further refines chin position and facial balance. This report describes the management of a young adult patient presenting with facial asymmetry due to unilateral mandibular hypoplasia. Clinical and radiographic examination revealed reduced ramal height on the affected side, deviation of the chin toward the hypoplastic side, and occlusal canting. A staged surgical approach was planned. In the first phase, intraoral DO was performed at the mandibular ramus-body region. In the second phase, genioplasty was performed to correct residual chin deviation and improve lower facial harmony. Postoperative results showed significant improvement in facial symmetry, mandibular length, and chin alignment.

## Introduction

Facial symmetry plays a major role in aesthetics, functional efficiency, and psychosocial well-being [1]. The mandible contributes significantly to lower facial contour, occlusion, and airway support. Any disturbance in mandibular growth may lead to asymmetry, functional problems, and compromised facial harmony [2].

Mandibular asymmetry commonly occurs due to unilateral condylar hypoplasia, trauma, infection, or temporomandibular joint (TMJ) ankylosis during the growing years [3,4]. The affected side often shows reduced ramal height, a deep antegonial notch, and chin deviation toward the side affected [2]. In long-standing cases, compensatory dental changes and occlusal canting may also develop.

Distraction osteogenesis (DO) has emerged as a reliable technique for skeletal lengthening. It promotes gradual bone formation while allowing adaptive changes in muscles, nerves, vessels, and soft tissues [1]. Once skeletal symmetry is improved, genioplasty can be used to fine-tune chin position and enhance facial balance [1].

This report describes the staged management of unilateral mandibular asymmetry using DO, followed by extended orthomorphic genioplasty.

## Case presentation

A 22-year-old male patient reported to the Department of Oral and Maxillofacial Surgery with the chief complaint of facial asymmetry and deviation of the chin since adolescence. He was concerned about his facial appearance and reported difficulty in chewing and speech.

There was no clear history of trauma, but he recalled a childhood episode of facial infection. No previous surgical treatment had been undertaken.

Clinical examination

Extra-oral Findings

**Figure 1:**
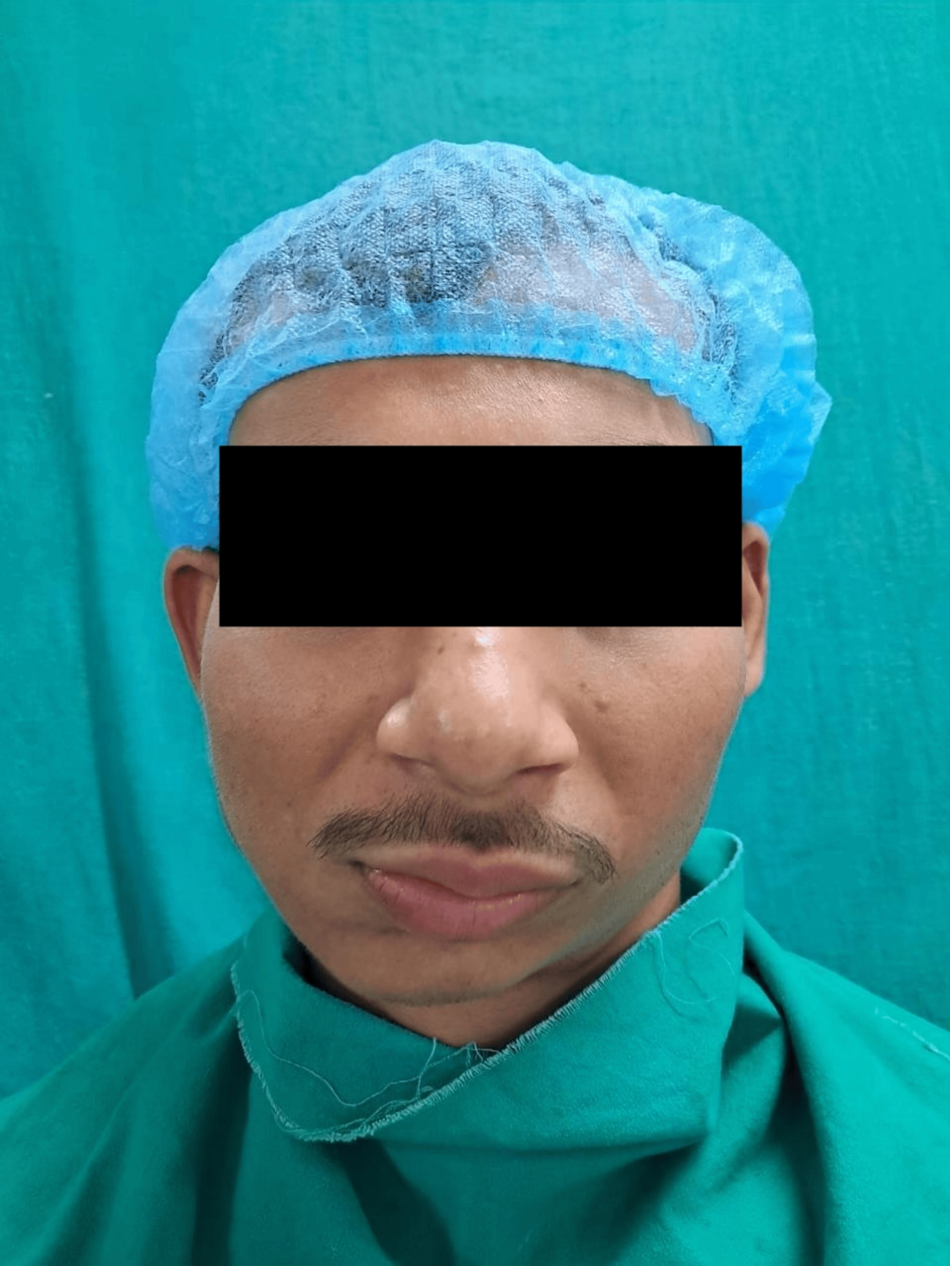
Preoperative extra-oral view of the patient

Extra-oral examination revealed noticeable facial asymmetry, a chin deviated toward the right side, reduced lower facial height on the affected (right) side, flattening of the mandibular angle region, and mild occlusal cant (Figure 1).

On palpation, the ramus on the affected side appeared shorter. Mouth opening was adequate (approximately 38 mm), and no active TMJ pain was noted.

Intra-oral Findings

Intra-oral examination showed crowding of lower anterior teeth (especially on the right side), crossbite tendency on the affected side, and occlusal plane cant.

Radiographic examination

**Figure 2:**
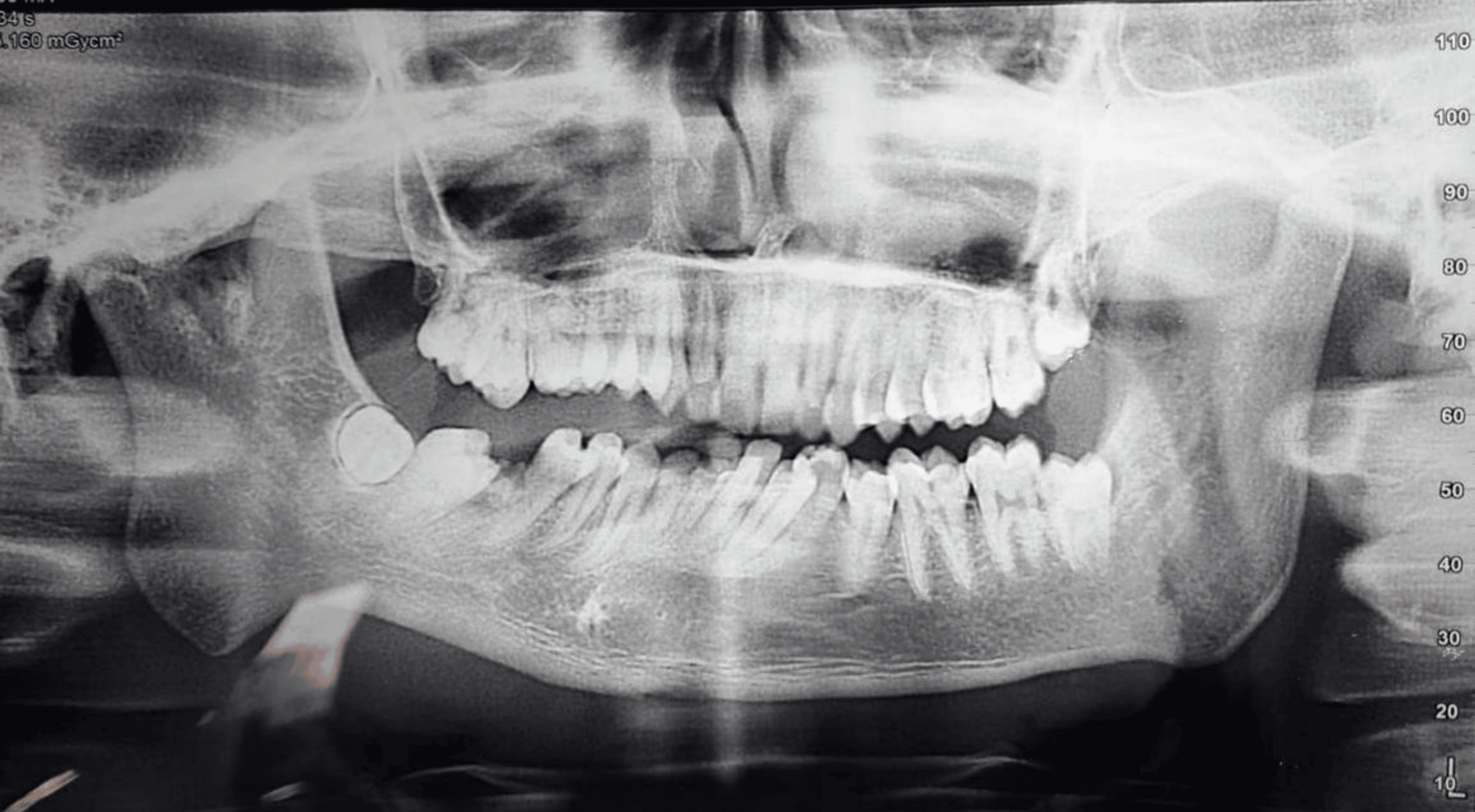
Preoperative orthopantomogram of the patient

Orthopantomogram (OPG) revealed (Figure 2) shortened ramus height on the right side, deep antegonial notch, and condylar deformity suggestive of hypoplasia.

**Figure 3:**
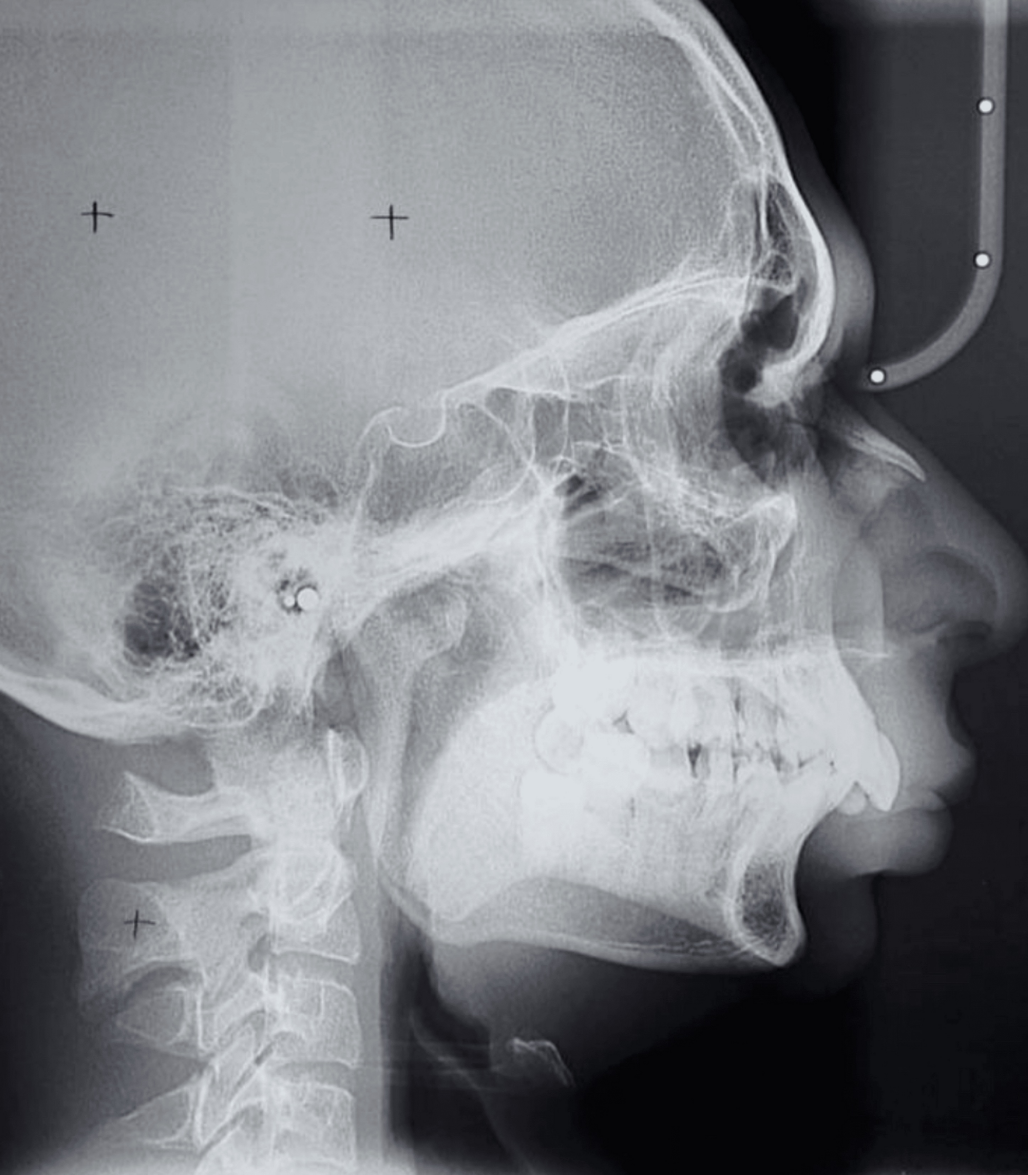
Preoperative lateral cephalometric radiograph of the patient

Lateral cephalometric (Figure 3) analysis showed skeletal discrepancy with mandibular retrusion on the right side. Three-dimensional imaging confirmed asymmetrical mandibular body length and ramal height deficiency. Based on clinical and radiographic findings, a diagnosis of unilateral mandibular hypoplasia leading to facial asymmetry was made.

Treatment planning

The objectives of the treatment were to enhance aesthetic appearance by improving facial symmetry along with increasing ramal height on the affected side, correcting chin deviation, and achieving stable skeletal correction.

Considering the amount of skeletal deficiency, conventional orthognathic advancement was not preferred due to potential relapse and limited soft tissue adaptability. A two-stage surgical approach was planned: stage 1 was DO of the affected mandibular ramus, and stage 2 was extended orthomorphic genioplasty for final chin correction.

Surgical procedure

Stage 1: Distraction Osteogenesis

**Figure 4:**
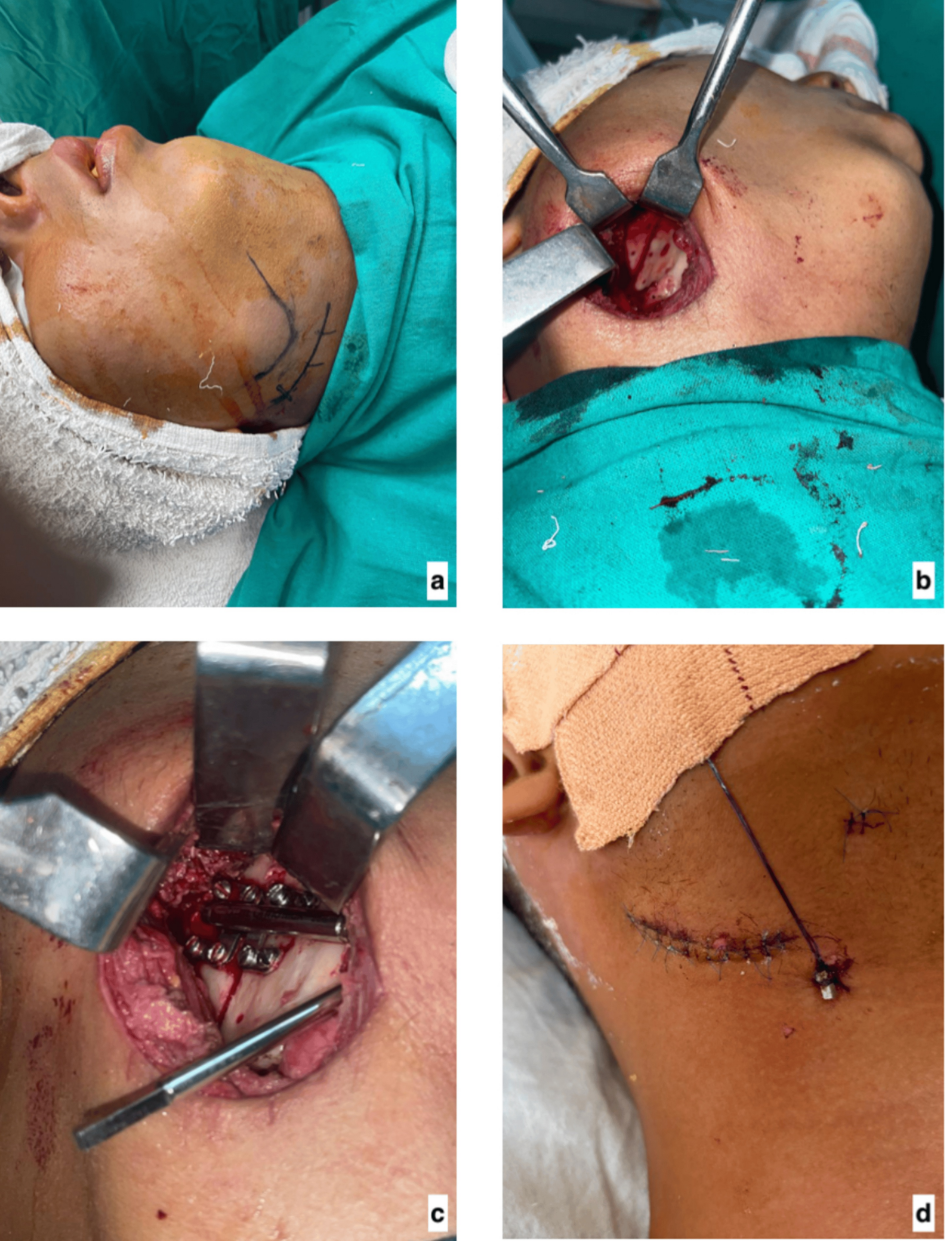
Intraoperative pictures of the distraction osteogenesis a. Marking the preferable incision line. b. Corticotomy in the ramus-body region. c. Placement of the internal distractor. d. During the latency period.

Under general anesthesia with nasotracheal intubation, an extraoral submandibular approach (Figure 4a) was used to expose the mandibular angle and ramus region. Care was taken to protect the facial artery, veins, and marginal mandibular nerve.

A corticotomy was performed in the ramus-body region while preserving the lingual periosteum to maintain blood supply (Figure 4b). A unidirectional internal distractor device was adapted and secured using titanium screws (Figure 4c).

After wound closure, a latency period of five days was observed to allow initial callus formation (Figure 4d).

Distraction phase: Activation began on the sixth postoperative day at a rate of 1 mm per day (0.5 mm twice daily). Gradual elongation was continued for 14 days until the desired vertical and horizontal correction was achieved. The patient was reviewed weekly during activation. Progressive improvement in chin alignment was clinically visible.

Consolidation phase: The distractor was left in place for 10 weeks to allow mineralization of the newly formed bone. Radiographic evaluation confirmed satisfactory bone formation within the distraction gap.

Stage 2: Extended Orthomorphic Genioplasty

**Figure 5:**
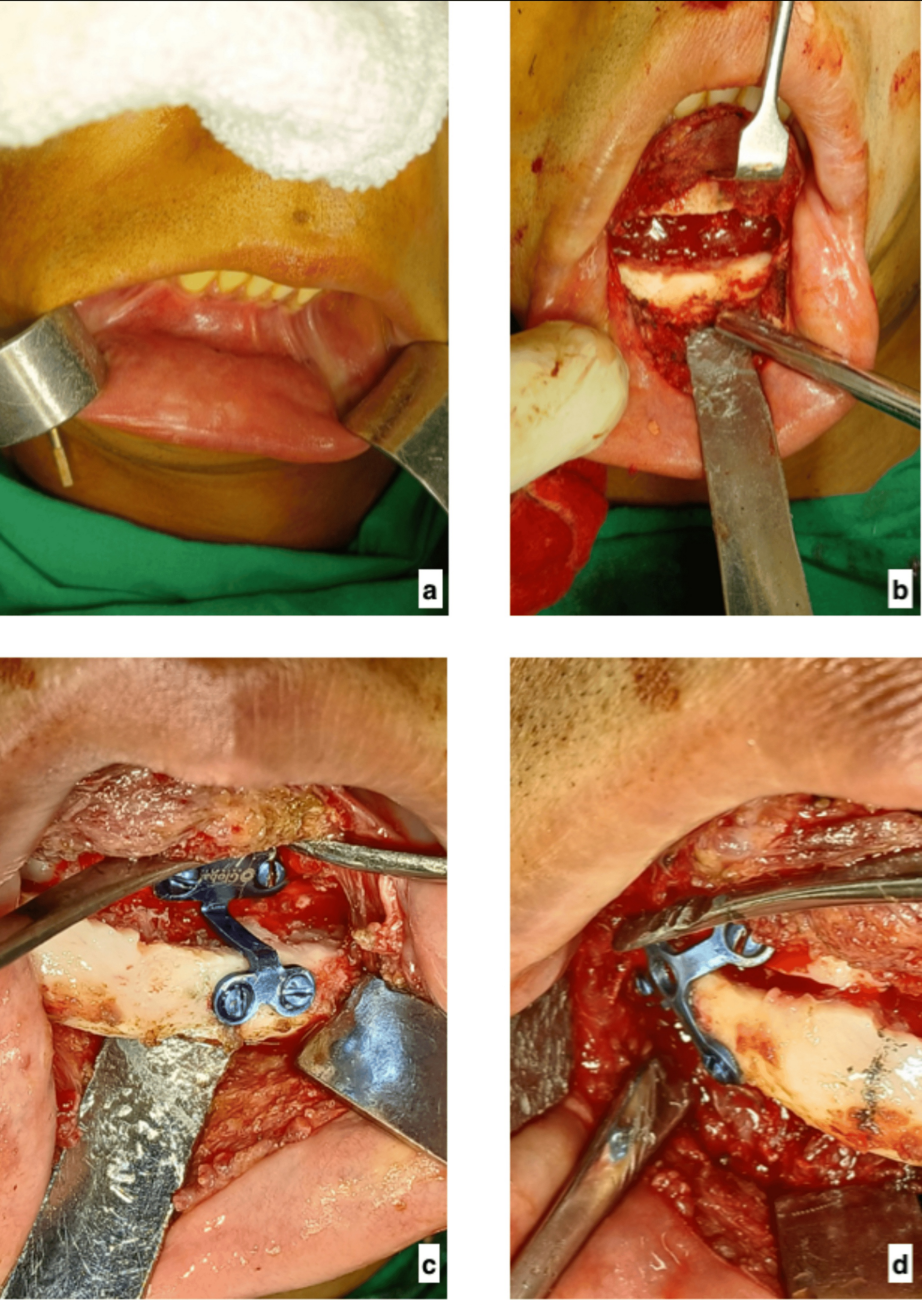
Intraoperative pictures of genioplasty a. Marking of the lower vestibular incision line. b. Osteotomy and repositioning of the chin. c. Segment fixed with conventional chin plate. d. Segment fixed with 'X' shaped plate.

After consolidation and distractor removal, residual chin deviation was still noticeable despite the correction of ramal height. Therefore, extended orthomorphic genioplasty was planned. Under general anesthesia, a lower vestibular incision (Figure 5a) was made to expose the chin region. A carefully designed osteotomy was performed, extending beyond the mental foramen to allow three-dimensional repositioning. The mobilized chin segment was repositioned: advanced anteriorly, shifted toward the midline, and rotated slightly to correct asymmetry (Figure 5b).

Semi-rigid fixation was achieved using two titanium plates (one is a conventional chin plate and the other is X-shaped) and screws (Figure 5c, 5d). The surgical site was closed in layers.

Postoperatively, antibiotics and analgesics were prescribed, a soft diet was advised for two weeks, and regular follow-up visits were scheduled. Postoperative swelling subsided within two weeks. No neurosensory deficit was reported.

**Figure 6:**
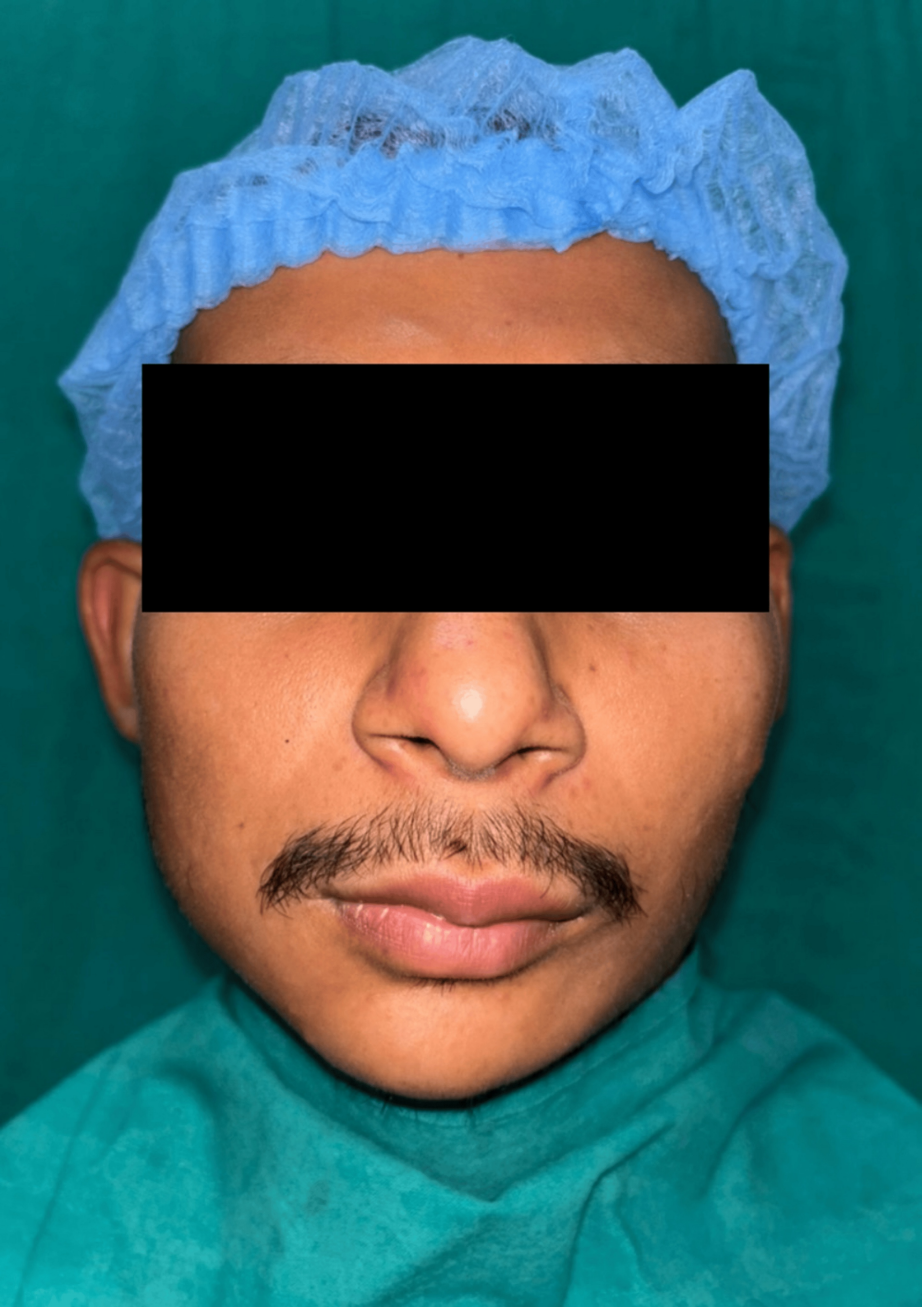
Postoperative extra-oral photograph in the third month

**Figure 7:**
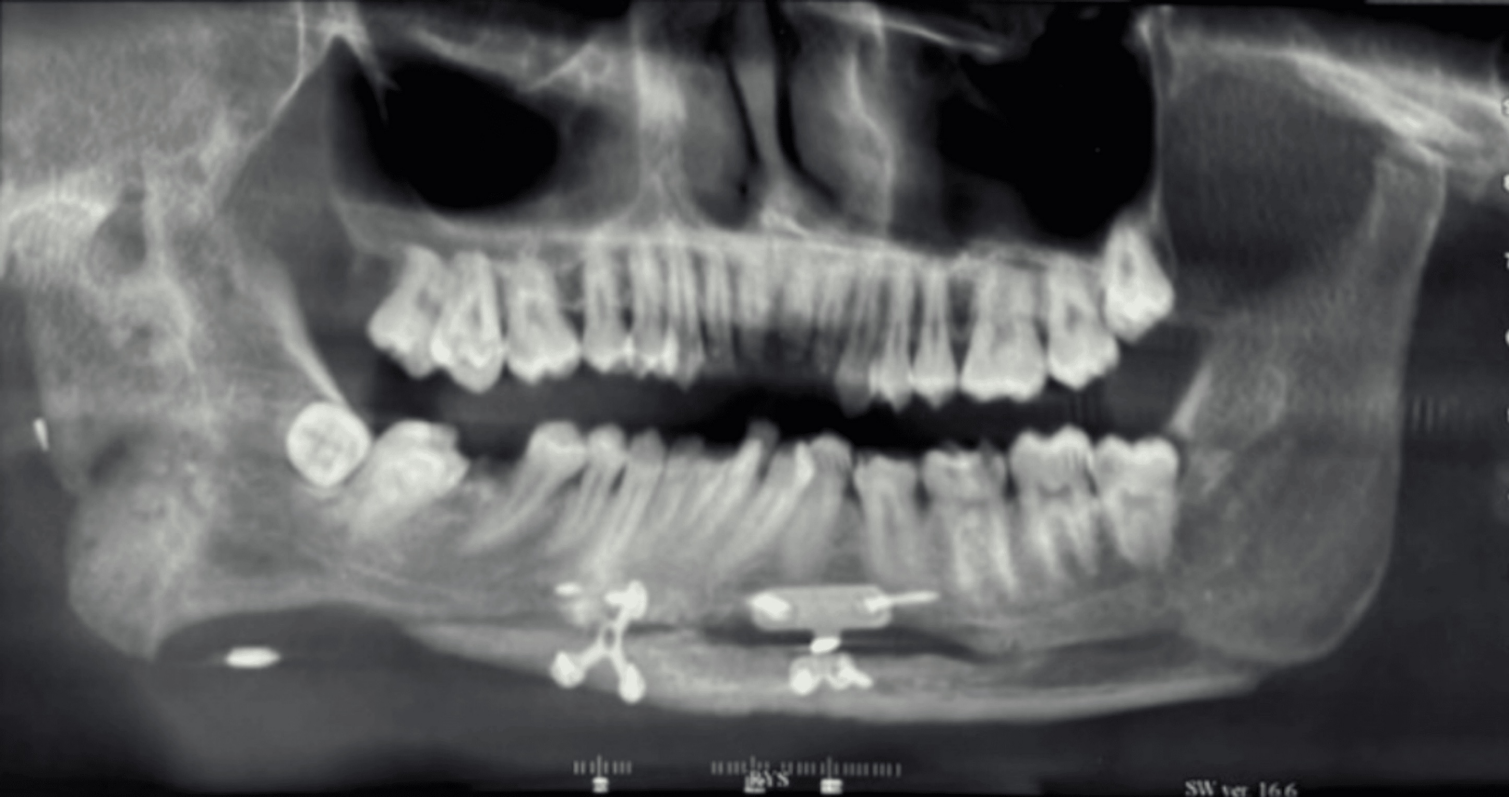
Postoperative cone beam computed tomography radiograph (coronal view) in the third month

At three-month follow-up: Facial symmetry was significantly improved, the chin was aligned with the facial midline, occlusal cant was reduced, and the patient was highly satisfied (Figure 6). A radiograph was taken (Figure 7).

Radiographs showed stable bone formation and satisfactory healing of the genioplasty segment. No relapse or complications were observed during the one-year follow-up.

## Discussion

DO was first invented by Codivilla (1905) and popularised by Ilizarov (1950) [5,6]. In the craniofacial region, the first application of DO was by McCarthy et al. [7]. DO is based on the principle of gradual mechanical traction, stimulating new bone formation between separated segments. First, callus formation occurs between cut bony fragments, and as traction is given, callus tissues also get stretched, which helps in the formation of new bone [8]. Unlike conventional osteotomy, DO allows simultaneous adaptation of soft tissues such as muscles, nerves, skin, and mucosa [9,10]. This reduces relapse risk and permits correction of larger discrepancies.

In unilateral mandibular hypoplasia, vertical ramal deficiency is a major component of asymmetry. Lengthening the ramus restores mandibular height and improves occlusal plane orientation. Gradual distraction also minimizes sudden stretching of surrounding tissues [11,12].

However, distraction alone may not completely correct chin deviation, especially in long-standing asymmetry. The chin position is influenced by both ramal height and symphyseal morphology. Therefore, genioplasty plays a crucial role in final aesthetic refinement [13].

Extended orthomorphic genioplasty allows multi-directional correction. It can address asymmetry, vertical deficiency, and retrusion simultaneously. When combined with DO, it produces a harmonious lower facial contour [3,14].

This staged approach provides several advantages: better soft tissue adaptation, reduced relapse, improved airway support, and enhanced aesthetic outcome.

Careful planning, accurate vector control, and patient compliance during activation are essential for successful results.

## Conclusions

Mandibular asymmetry resulting from unilateral hypoplasia can be successfully corrected by a carefully planned combination of DO and extended orthomorphic genioplasty. DO not only restores mandibular length and symmetry but also allows gradual adaptation of surrounding soft tissues, reducing the risk of relapse and improving functional outcomes. The subsequent genioplasty plays a crucial role in fine-tuning chin position, enhancing facial balance, and achieving optimal aesthetic results. A staged, individualised treatment plan, meticulous surgical technique, and postoperative follow-up are essential for achieving predictable and stable outcomes. Overall, this combined approach offers a reliable and effective result, leading to significant improvement in both function and facial harmony, thereby enhancing patient confidence and quality of life in the long term.
